# Treatment of delayed union of the forearm with extracorporeal shockwave therapy: a case report and literature review

**DOI:** 10.3389/fendo.2023.1286480

**Published:** 2023-11-15

**Authors:** Larisa Ryskalin, Federica Fulceri, Gabriele Morucci, Stefania Dell’Agli, Paola Soldani, Marco Gesi

**Affiliations:** ^1^ Department of Translational Research and New Technologies in Medicine and Surgery, University of Pisa, Pisa, Italy; ^2^ Center for Rehabilitative Medicine “Sport and Anatomy”, University of Pisa, Pisa, Italy; ^3^ Independent Researcher, Livorno, Italy

**Keywords:** extracorporeal shockwave therapy, delayed union fracture, bone healing, long bone fracture, forearm

## Abstract

Compared to other long bones, forearm fractures are particularly challenging due to the high rate of complications. These include malunion, delayed/nonunion, wrist and elbow movement reduction, and pain. Surgical procedure is considered the gold standard for managing delayed union and nonunion of the long bones. However, in the last decades, extracorporeal shockwave therapy (ESWT) has emerged as an effective and less invasive approach to enhance bone regeneration and fracture healing, avoiding major complications of surgical procedures. In contrast to the broad literature reporting good clinical results of ESWT in the treatment of nonunions, there is currently limited evidence regarding the clinical application of shock waves on long bone delayed fractures, particularly those of the forearm. In the present paper, we report a case of delayed bone healing of the diaphyseal region of the ulna treated with focused ESWT. The successful case experienced bone healing at the fracture site in less than 3 months after initial ESWT treatment. Acknowledging the limitation of reporting a case report, however, the remarkable clinical results and the absence of side effects contribute valuable information in support of the use of ESWT as an effective alternative to standard surgery for forearm fractures.

## 1 Introduction

Physiological fracture healing occurs within 3 months after bone injury hrough an intricate and highly coordinated regenerative process (1). However, several local and/or systemic factors can contribute to retardation or failure of bone consolidation (2). As a result, up to 10% of patients with long bone fractures suffer from healing complications, which include both delayed and nonunion (1, 3). In particular, a delayed union is defined as the absence of radiological progression of healing 3 months after the initial injury, whereas nonunion is considered when the fracture fails to unite over 6 months (4, 5). This, in turn, has several clinical complications that can lead to patients’ reduced mobility in daily activities and working capacities, reduced quality of life, and increased healthcare costs (6).

Albeit conventional surgery intervention represents the gold standard for treating delayed unions and nonunions, in the last decades less invasive approaches have been implemented to enhance bone regeneration and fracture healing while avoiding hazards and complications of surgical interventions (2, 7). In this regard, delayed unions require careful evaluation, as this can change their clinical course and management. In fact, delayed unions may result in further surgery with subsequent prolonged or repeat hospitalization. This, in turn, may prolong patient’s disability, and delay his return to the workforce, while adversely impacting his quality of life (4). Thus, if a delayed union is suspected, less invasive treatments may be tried at first, before pursuing major surgery. These include electromagnetic stimulation (8), electrical capacitive coupling (9), low-intensity pulsed ultrasound (10), or other biological stimulation methods such as bone autograft and cell-based therapies (11).

Within this frame, the use of ESWT has gathered increasing attention due to its biological potential in enhancing osteogenesis (12, 13) and thus promoting fracture healing (5, 7, 14).

Increasing evidence in basic research demonstrates that shock wave stimulation generates its effect in tissue via mechanotransduction which triggers several endogenous bone regeneration processes via cell proliferation, differentiation, and migration (15–17). Furthermore, there are several clinical observational studies on the beneficial effects of ESWT on bone healing (7, 13, 14, 18, 19). For instance, a very recent systematic review of the literature conducted on three main databases (i.e., PubMed, Scopus, and Web of Science) showed that out of 1200 total long bone nonunions, 876 (73%) healed after being treated with ESWT, with hypertrophic cases achieving 3-fold higher healing rates when compared to oligotrophic or atrophic cases (14). Again, another recent retrospective study reported positive outcomes, defined by radiographic bone consolidation 6-month follow-up and absence of both pain and functional limitations during normal weight loading, in 16 out of 22 (73%) patients treated with rESWT for fracture nonunions that failed to heal despite initial surgical fixation (13). Although the healing rates achieved with surgery are sometimes comparable to those of ESWT treatments, however these latter do not carry any risk of possible complications.

Compared to the substantial body of current literature supporting the use of shock waves in the treatment of long bone nonunions, there is little evidence concerning the efficacy of high-energy ESWT for the treatment of delayed fractures. Furthermore, most of these studies concern the delayed union of the long bones of the lower limbs, as well as metatarsal and scaphoid fractures (20).

To our knowledge, there is currently little evidence of the treatment of the ulnar delayed unions of the diaphyseal region with ESWT. Among forearm fractures, isolated diaphyseal fractures of the ulna, without an accompanying radius injury, are fairly rare. Moreover, forearm fractures show a high complication rate including malunion, nonunion, reduction in the range of wrist and elbow movements, and pain (21). Indeed, the management of forearm bone fractures is particularly challenging because the two bones (i.e., the ulna and the radius) act in a particular way in the prono-supination phenomenon and several key muscles assisting prono-supination may exert deforming forces leading to long-term forearm disability if neglected (22). Therefore, timely and accurate management of these patients is pivotal in gaining optimal functional outcomes, preserving upper limb function, as well as minimizing complications.

Here, we report the promising outcomes of a delayed ulnar fracture treated with focused high-energy ESWT.

## 2 Case presentation

**Figure 1:**
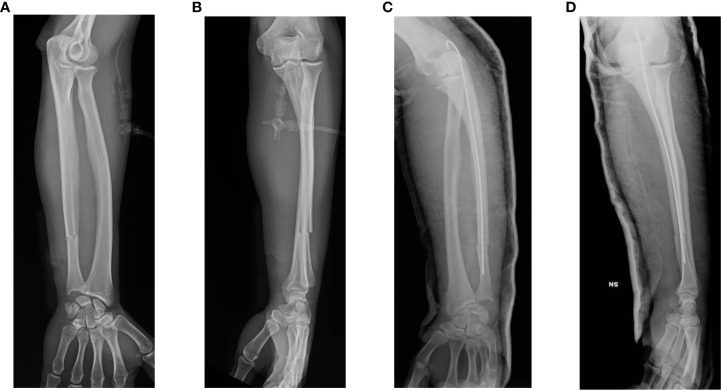
Pre- and post-operative imaging of the patient’s left forearm fracture. Preoperative anterior–posterior **(A)** and lateral **(B)** radiographs show the complete fracture of the left distal ulna. Postoperative anterior-posterior **(C)** and lateral **(D)** X-rays the day after internal fixation surgery.

A 28-year-old, right-dominant handed man, involved in a road traffic accident has sustained an injury to his left forearm resulting in an isolated distal-third fracture of the ulna (**Figures 1A, B**). Due to the occurrence of a concomitant contused lacerated wound at the level of the volar aspect of the ulna, within the next 24h, the fracture was fixed and stabilized with percutaneous intramedullary Kirschner wire (K-wire), inserted through the olecranon in a proximal-distal direction (**Figures 1C, D**). The post-operative X-ray was satisfactory, with no sign of immediate surgical complications.

**Figure 2:**
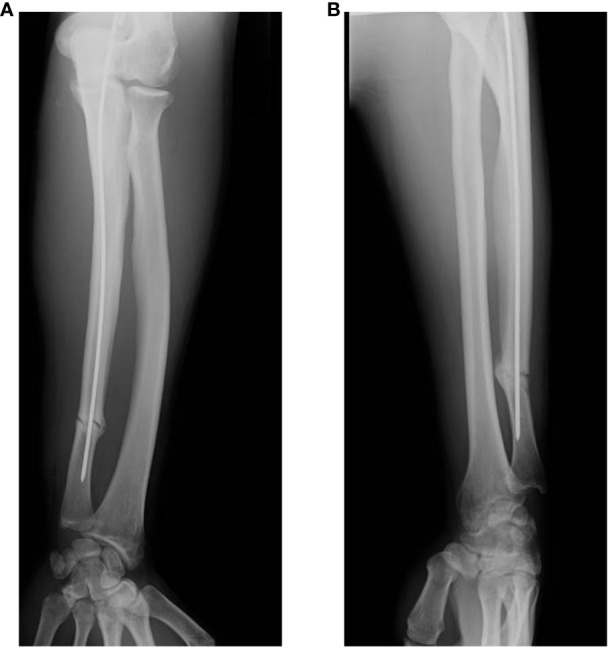
Radiographs at 3 months after surgery. Fracture consolidation is still not achieved as shown by anterior–posterior **(A)** and lateral **(B)** X-rays of the left ulna.

The patient was discharged from the hospital after 72 h of observation with no sign of peripheral neuro-vascular injury associated with the bone fracture. The patient was advised by the orthopedic surgeon to keep the forearm immobilized with a splint, to keep unloaded the arm, and to avoid straining and weightlifting with his left hand. Radiological assessment was the primary outcome, and it was performed at different time points (i.e., monthly) to monitor fracture healing. However, over 3 months after surgery, X-ray imaging showed no osteogenesis and absence of bone union at the fracture site. Thus, a delayed bone union was diagnosed (**Figure 2**).

At that time, the patient presented himself at the Center for Rehabilitative Medicine “Sport and Anatomy” of the University of Pisa and a series of shock wave sessions was started. In detail, high-energy focused ESWT (f-ESWT) was performed at the fracture site using a DUOLITH^®^ SD1 ultra (Storz Medical AG., Tägerwilen, Switzerland); no local anesthesia was applied. The patient underwent two cycles of treatments, at 3 weeks intervals, each one consisting of 5 and 4 sessions per cycle, respectively. Each f-ESWT session was performed once a week, with an average of 3,500 pulses at a 4.5 Hz frequency. The average energy flux density (EFD) was 0.25 mJ/mm^2^, depending on the patient’s pain tolerance limit. Total energy was 25.000 mJ per session on average (**Supplementary Table 1**). Treatment success was monitored with radiographs and clinical examinations. During both cycles of f-ESWT, no side effects (i.e., bruising or swelling at the treatment site, slight reddening of the skin, or transient local hematoma) were observed.

**Figure 3:**
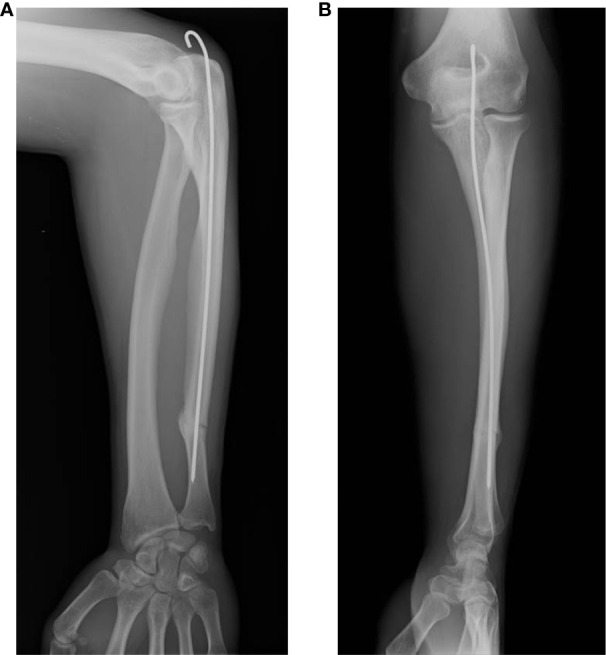
Radiographic consolidation at the fracture site after ESWT treatment. The presence of callus is visible on anterior–posterior **(A)** and lateral **(B)** X-rays of the left ulna.

Eleven weeks after f-ESWT, x-ray examination showed callus formation at the fracture site (**Figure 3**), as well as evidence of full bony healing in the further follow-up controls. Functional improvements in the affected limb were also observed after the second ESWT treatment. In addition, no pain or limited range of motion was observed, and the patient was able to return to daily life and work activities at full capacity.

## 3 Discussion

Diaphyseal fractures of the long bones of the forearm are commonly encountered in orthopedics and traumatological clinical practice and their management is still challenging (23, 24). Compared to other long bone fractures, those of the forearm are relatively complex, and proper treatment intervention is crucial to completely restore upper limb functions (25). Furthermore, several key muscles that assist forearm pronation/supination (i.e., pronator teres and pronator quadratus/biceps brachii and supinator, respectively) may exert deforming forces upon fracture fragments leading to forearm deformities (22), and thus significant disability.

To date, surgical approaches remain the gold standard to achieve anatomic fracture reduction, stable fixation, and functional preservation, thereby enabling patients to return to daily life activities as early as possible. However, the invasiveness of these approaches can negatively interfere with the clinical outcomes often leading to serious complications such as infections, peripheral nerve injury, persistent pain, malunion, and nonunion (24).

In an effort to achieve bony union more rapidly and in a non-invasive way, over the last decade, EWST has emerged as a promising alternative to surgery. Pioneer studies on dog and rabbit nonunion models showed the effectiveness of ESWT in promoting callous formation (18, 26, 27), enhancing recovery of the mechanical properties of the bone (28, 29), as well as increasing union rates (30, 31). In line with this, a recent paper showed that ESWT might accelerate endochondral ossification and bone formation in a rat femur delayed-union model (32). Since then, the beneficial effect of ESWT for nonunion fractures of long bones has been reported in several experimental and clinical studies (7, 13, 14, 28–33). According to some reports, ESWT is also recommended as a first treatment choice for delayed bone healing (16, 34–37) or pseudarthrosis (20, 38–40). Nevertheless, when analyzing more in-depth the current literature, it appears less supportive of the ESWT-induced bone healing process for delayed unions. Indeed, contrary to the broad experience of ESWT treatment for nonunion fractures, there is a lack of sufficient amount of data regarding delayed unions.

Despite preliminary clinical data reported studies demonstrating good clinical results for ESWT in delayed union fractures, the results (though all positive) greatly varied among the studies with ratings of success ranging from 50% up to 80% (41). For instance, in 2010, Zelle et al. reviewed 10 clinical studies and found that the overall union rate in patients with delayed union/nonunion was 76% (95% confidence interval 73%-79%), ranging from 41% to 85% (42). In another recent literature review on delayed fracture healings, Willems et al. found an average union rate after ESWT of 86% (5). This, in turn, may be due to the variability of treatment protocols and/or the limited methodological quality of these studies. Some authors argued about deficiencies in the study design of most previously published studies (5, 42–44).

Therefore, we carefully revised the current literature in order to provide evidence for the effectiveness of ESWT in the treatment of delayed long-bone fractures of the forearm, and especially those of the ulna (**Table 1**). When analyzing the literature, it emerges that the anatomic fracture localization of delayed unions is quite heterogeneous within studies, with the long bones of the lower extremity (i.e., femur and tibia) being the most affected ones. With reference to the upper limb, the scaphoid bone is the most frequently fractured one. However, most of the studies do not separate the results for delayed unions from those of nonunions (14, 36, 43, 45, 46). At the same time, in some previous publications, the precise localization of delayed fracture is not always described. For instance, Schaden et al. (35) reported the successful use of ESWT in the treatment of over 3,500 delayed healing fractures and pseudarthroses with an average success rate of almost 80% after six months of follow-up, without indicating the different fracture locations. In the paper by Biederman et al. (43), patients with delayed bone healing showed a higher and earlier rate of union (93%; mean time to union, 3.4 months; range, 0.2–4.9 months) compared with patients with nonunion. However, the study does not specify the site of delayed unions, rather it reports “long bones and others” in a quite general way. Similarly, in another paper, it is not indicated whether the 349 specific bones treated with ESWT were associated with a delayed or fracture nonunion (45).

**Table 1:** Evidence for ESWT application for delayed unions of the forearm.

Refs.	DU (no.)	Localization	Time from injury/ diagnosis and ESWT	Time to union (mo.)	ESWT device	No. of shocks per session	EFD (mJ/mm^2^)	Healing results (success rate %)
(2)	9	Long bones and others	≤181 days	3 to 6 mo.	LithoSpaceOrtho	3000	0.36	8/9 (88.8%)
(37)	42	Long bones *	NR	3 to 6 mo.	Econolith 2000 lithotripter	1500-3000 (20 kV)	NR	40/42 (95%)
(36)	35	Long bones and others *	3 to 6 mo.	NR	NR	1000-12000^	0.25-0.4	26/35 (74.3%)
(43)	16^#^	Long bones (n=13)^§^ Others (n=1)	5±3 mo.	3.4±1.4	Electrohydraulic MFL 5000 Lithotriptor	2900 (23 kV)	0.7	12/13 (93%)
(45)	120	Long bones and others *	≤181 days	NR	Orthowave 280	4000-12000 (26-28 kV)	0.38-0.40	102/120 (85.0%)
(46)	9	Long bones and others *	71.33 weeks (for successful DU)	NR^‡^	Electrohydraulic lithotripter Econolith 2000	3000 (20-21 kV)	NR	4/9 (44.4%)

DU (no.), number of delayed union; mo., months; EFD, Energy flux density; NR, Not reported; * The anatomic localization of delayed union is not specified; ^ Shock wave intensity and number of shock waves were selected according to the area of the fracture gap and the cross section of the bone to be treated (Scaphoid: 0.25 to 0.35 mJ/mm2 (20–24 kV), 1000–2500 shock waves; tibias and femurs: 0.4 mJ/mm2 (28 kV), 12,000 shock waves); # Two patients with delayed metatarsal stress fractures refused radiographic controls, as they were free of complaint 6 weeks after therapy; § The Authors do not specify whether delayed union occurred in upper or lower limbs; ‡ follow-up at 24 weeks.

Another key point is that there is a high variability in the definition of “delayed union” which is not homogeneous among the studies. For instance, some Authors defined delayed unions as fractures that do not show radiological union 3 months after fracture (5). In the paper by Schaden et al. (36), the delay from the initial injury or the last operation was 3 to 6 months (delayed healing). Otherwise, in other papers, the Authors included those fractures that showed no progressive callus formation as well as the absence of radiographic progression of healing upon clinical examination by six months after injury (2, 4, 43, 45, 47). However, this may be due to the fact that there is no clear consensus among orthopedic surgeons in the assessment of fracture healing based on clinical evaluation and radiological examinations (47–49).

Again, there is a lot of heterogeneity in the treatment protocols for delayed unions between the studies, both in terms of ESWT devices, number of sessions, number of shock waves per session, total energy flux density, and so on. This, in turn, might be another explanation for those divergent healing rates.

Despite all the limitations reported above regarding previous literature, in any case, it is important to underline that no adverse severe effects (i.e., neuromuscular, systemic, or device-related local complications) have been reported, which strongly suggests that ESWT is a safer alternative option to surgical treatment of delayed union and nonunions (5, 19, 37, 50). Remarkably, in a very recent paper, Dahm et al. reported that older age and fracture localization in the diaphysis or distal metaphysis of the humerus represent negative predictive factors for a successful ESWT outcome (47). In fact, the largest late healing effects between the 3- and 6-month follow-up were found for humeral diaphysis compared to other anatomical regions, such as the proximal metaphyseal localization of the lesion. Data reported in the present case report are encouraging since with our treatment protocol we achieved bony consolidation of the diaphyseal region of the ulna in less than 3 months after the first ESWT treatment. Besides anatomic fracture location, the time to the shockwave therapy following the injury may negatively impact healing outcomes (45). In particular, concerning the ulnar bone, the estimated probability of a positive fracture-healing at < 181 days between injury and ESWT therapy is 80.0%, whereas it significantly deteriorates down to 64.9% when more than eleven months (339 days) elapsed between the injury and first ESWT treatment exceeds (45).

## 4 Conclusions

The good clinical results and the absence of side effects reported in the present study suggest that ESWT should be considered a valid noninvasive treatment option for stimulating bone healing for delayed fractures of the ulnar bone.

Acknowledging the limitation of a case report, however, this paper contributes valuable information. In fact, according to our data, it emerges how the timeliness of an adequate diagnosis and early ESWT therapeutic approach is pivotal in avoiding unfavorable evolution of the delayed fracture unions, which are configured with functional limitations and patient disability.

Further randomized, prospective clinical trials are needed to standardize both the healthcare decision-making as well as the optimal site-specific ESWT protocol for the treatment of delayed and non-healing fractures.

## Data availability statement

The raw data supporting the conclusions of this article will be made available by the authors, without undue reservation.

## Ethics statement

Ethical review and approval were waived for this treatment since it was part of ordinary clinical activity. The studies were conducted in accordance with the local legislation and institutional requirements. The participants provided their written informed consent to participate in this study. Written informed consent was obtained from the individual(s) for the publication of any potentially identifiable images or data included in this article.

## Author contributions

LR: Conceptualization, Data curation, Formal analysis, Methodology, Writing – original draft, Writing – review & editing. FF: Conceptualization, Formal analysis, Methodology, Writing – original draft, Writing – review & editing. GM: Writing – review & editing. SD: Formal analysis, Writing – review & editing. PS: Supervision, Writing – review & editing. MG: Conceptualization, Funding acquisition, Methodology, Supervision, Writing – review & editing.
