# Supplementary material for: Treatment of delayed union of the forearm with extracorporeal shockwave therapy: a case report and literature review
Source: Front Endocrinol (Lausanne). 2023 Nov 15;14:1286480. doi: 10.3389/fendo.2023.1286480 (PMC10684947; doi:10.3389/fendo.2023.1286480)
Supplement: Supplementary file 1 [file Table_1.docx]

**Supplementary Table 1.** ESWT treatment protocol.

| **Type of**  **ESWT** | **Device/principle** | **No. of treatments**  **(i.e. cycles)** | **no. of session**  **per treatment** | **Pulses per**  **session** | **EFD**  **(mJ/mm^2^)** | **Total energy**  **per session** |
| --- | --- | --- | --- | --- | --- | --- |
| Focused | Duolith SD1 ultra, Storz/  Electromagnetic | 2 with  3-wk interval | 5 sessions (1^st^ cycle)  + 4 sessions (2^nd^ cycle) | 3.500 pulses/session  (4.5 Hz frequency) | 0.25 mJ/mm^2^ | 25.000 mJ |
